# Photodynamic Inactivation of Methicillin-Resistant *Staphylococcus aureus* by a Natural Food Colorant (E-141ii)

**DOI:** 10.3390/molecules25194464

**Published:** 2020-09-29

**Authors:** Cynthia S. A. Caires, Cicera M. Silva, Alessandra R. Lima, Lurian M. Alves, Thalita H. N. Lima, Ana C. S. Rodrigues, Marilene R. Chang, Samuel L. Oliveira, Corinne Whitby, Valter A. Nascimento, Anderson R. L. Caires

**Affiliations:** 1 Graduate Program in Health and Development in the Midwest Region, Faculty of Medicine, Federal University of Mato Grosso do Sul, Campo Grande 79070-900, Brazil; cynthiasuzyelen@yahoo.com.br (C.S.A.C.); anaclaurod@yahoo.com.br (A.C.S.R.); marirchang@yahoo.com.br (M.R.C.); 2 School of Life Sciences, University of Essex, Colchester CO4 3SQ, UK; cwhitby@essex.ac.uk; 3 Optics and Photonics Group, Institute of Physics, Federal University of Mato Grosso do Sul, Campo Grande 79070-900, Brazil; ciceraquimica@gmail.com (C.M.S.); ramos.alessandra.09@gmail.com (A.R.L.); lurianalves@gmail.com (L.M.A.); thalita.lima212@gmail.com (T.H.N.L.); samuel.oliveira@ufms.br (S.L.O.)

**Keywords:** sodium copper chlorophyllin, E-141ii, photoinactivation, *Staphylococcus aureus*, MRSA, antimicrobial resistance

## Abstract

This study evaluates the photosensitizing effectiveness of sodium copper chlorophyllin, a natural green colorant commonly used as a food additive (E-141ii), to inactivate methicillin-sensitive and methicillin-resistant *Staphylococcus aureus* under red-light illumination. Antimicrobial photodynamic inactivation (aPDI) was tested on a methicillin-sensitive reference strain (ATCC 25923) and a methicillin-resistant *Staphylococcus aureus* strain (GenBank accession number Mh087437) isolated from a clinical sample. The photoinactivation efficacy was investigated by exposing the bacterial strains to different E-141ii concentrations (0.0, 1.0, 2.5, 5.0, 10.0, and 20.0 µM) and to red light (625 nm) at 30 J cm^−2^. The results showed that E-141ii itself did not prevent bacterial growth for all tested concentrations when cultures were placed in the dark. By contrast, E-141ii photoinactivated both methicillin-sensitive *Staphylococcus aureus* (MSSA) and methicillin-resistant *Staphylococcus aureus* (MRSA) under red-light illumination. However, different dose responses were observed for MSSA and MRSA. Whilst the MSSA growth was inhibited to the detection limit of the method with E-141ii at 2.5 µM, >10 µM concentrations were required to inhibit the growth of MRSA. The data also suggest that E-141ii can produce reactive oxygen species (ROS) via Type I reaction by electron transfer from its first excited singlet state to oxygen molecules. Our findings demonstrate that the tested food colorant has great potential to be used in aPDI of MRSA.

## 1. Introduction

The emergence of microbial drug resistance arises as a global challenge. A study estimates that about 700,000 people die every year for infections caused by drug-resistant strains of microorganisms [1]. Bacterial resistance to antibiotics is one of the major challenges to global public health of the 21st century [2] as the available antibiotics are becoming less effective due to their indiscriminate and increasing use in humans, animals, and plants [3,4]. Consequently, infectious diseases caused by multidrug-resistant bacteria bring back similar issues faced during the pre-antibiotic era [5,6]. Unless innovative actions are taken, the burden of deaths from antimicrobial multidrug resistance could rise to 10 million lives each year by 2050, with a cumulative cost of 100 trillion USD to global economic output [1,7].

Infections caused by *Staphylococcus aureus* are a serious health problem as *Staphylococcus aureus* is a major human pathogen which has the ability to acquire resistance to most antibiotics [8]. Nowadays, infections caused by methicillin-resistant *Staphylococcus aureus* (MRSA) are a challenge to public health worldwide because they are associated with high mortality rates when compared to ones induced by methicillin-sensitive *Staphylococcus aureus* (MSSA) strains [8]. It is estimated that MRSA-infected people are 64% more likely to die than those infected with MSSA [9]. MRSA has been the leading cause of healthcare-associated infections in Europe, with more than 171,000 nosocomial MRSA infections detected annually [10]. In the United States, MRSA causes at least 80,000 infections per year, resulting in approximately 11,000 deaths annually [11]. Therefore, it is necessary to propose and develop alternative ways to inactivate multidrug-resistant bacteria to avoid a devastating problem in the future [1]. Consequently, the World Health Organization (WHO) has indicated an urgent need to develop new antimicrobial medicines and treatments as a key objective in its global action plan on antimicrobial resistance [12].

In this scenario, antimicrobial photodynamic inactivation (aPDI) emerges as a promising alternative to conventional drugs [13,14]. It is based on the production of reactive oxygen species (ROS) by the interaction between molecular oxygen (O_2_) and a light-excited photosensitizer (PS), causing cell death [15]. Two types of reactions may occur during this interaction: (i) electron transfer from the excited triplet or singlet states of PS to O_2_, generating radical species such as O_2_^−^, OH^.^, and H_2_O_2_ (Type I); and (ii) energy transfer from the excited triplet state of PS to O_2_, producing singlet oxygen (^1^O_2_) by exciting the triplet ground state of oxygen (^3^O_2_) (Type II) [13,16,17,18].

**Figure 1:**
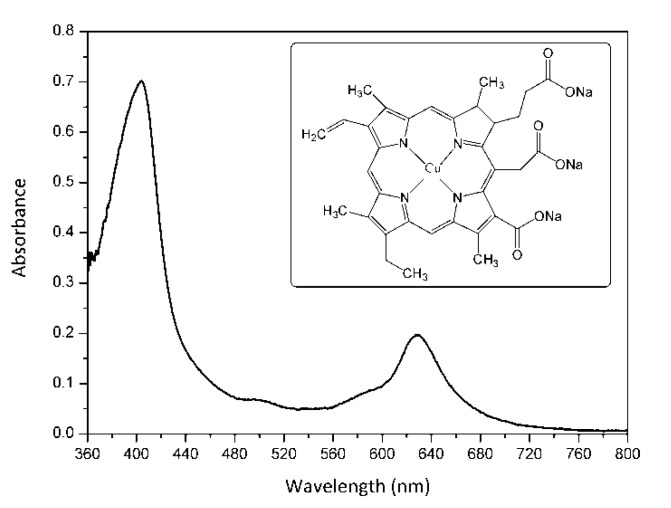
UV-vis absorption spectrum of E-141ii in distilled water. The inset shows its molecular structure.

The present study investigated an alternative way to inactivate in vitro MRSA strains based on aPDI and copper sodium chlorophyllin (E-141ii) as PS. E-141ii is a metal-chlorophyllin salt containing a paramagnetic Cu^2+^ center, which presents absorption bands in the blue and red regions related to the Soret and Q bands with maxima at around 404 and 628 nm, respectively (Figure 1). It is important to point out that E-141ii is a natural green colorant derived from chlorophyll authorized as a food additive in several countries (for instance, Europe, United States, and Brazil) [19,20,21,22]. As a food additive, E-141ii is a non-toxic compound, and it also presents a high extinction coefficient in the photodynamic therapy window (600–700 nm range), which is a desired feature of a good PS for medical applications [23]. Furthermore, in contrast to chlorophyll, E-141ii is water-soluble and stable to oxidation.

There are only a few reports on the antibacterial photoactivity of E-141ii [5,23,24,25,26,27,28,29]. Lopez-Carballo et al. tested the aPDI efficacy of E-141ii against Gram-positive and Gram-negative bacteria when incorporated into gelatin films [24]. Caires et al. showed that E-141ii subjected to red light efficiently inhibits the growth of Gram-positive bacteria [5]. Nevertheless, no aPDI effect was observed on Gram-negative bacteria. Despite being more effective against Gram-positive strains, it was demonstrated recently that E-141ii might be applied to photoinactivate Gram-negative bacteria when using a high PS concentration [26]. Although previous work has reported the application of E-141ii in aPDI of multidrug-sensitive Gram-positive bacteria [5,24,25], here, it is demonstrated for the first time that E-141ii can be successfully applied to photoinactivate MRSA strains. Moreover, the aPDI mechanism of action promoted by E-141ii is discussed.

## 2. Results and Discussion

**Figure 2:**
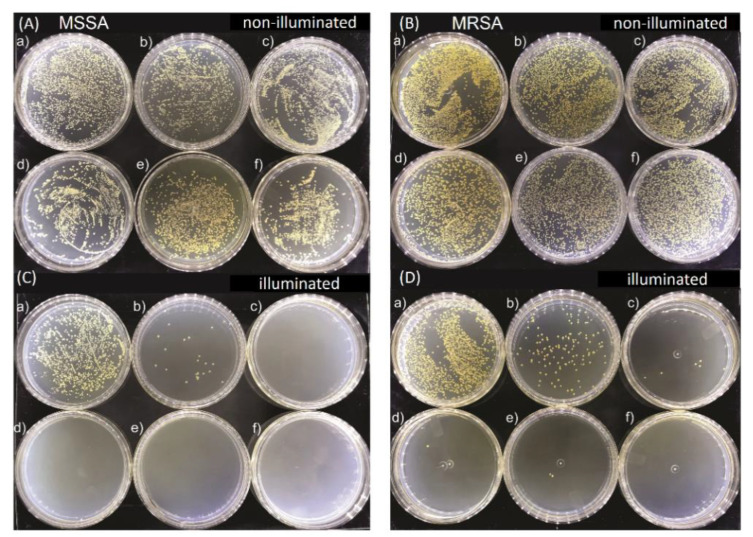
Growth of methicillin-sensitive *Staphylococcus aureus* (MSSA) (**A**) and (**C**), and methicillin-resistant *Staphylococcus aureus* (MRSA) (**B**) and (**D**) colonies in Petri dishes containing (**a**) 0.0, (**b**) 1.0, (**c**) 2.5, (**d**) 5.0, (**e**) 10.0, and (**f**) 20.0 µM of E-141ii. The illuminated groups were submitted to 625 nm light for 1 h (30 J cm^−2^).

**Figure 3:**
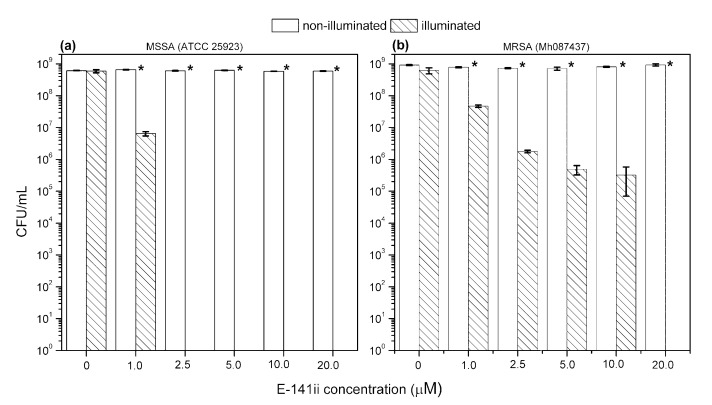
Growth response of MSSA (**a**) and MRSA (**b**) when submitted to different concentrations of E-141ii. The irradiated groups were illuminated at 625 nm for 1 h (30 J cm^−2^). * indicates a significant difference (paired sample *t*-test, *p* < 0.05) between the illuminated and non-illuminated groups when compared at a fixed E-141ii concentration. Error bars represent the standard deviation.

Figure 2 shows representative images of MSSA and MRSA colonies on plate count agar for all used E-141ii concentrations when subjected to the red-light irradiation and kept in the dark. The mean values (±SEM) of the CFU/mL, obtained from replicate measurements, are presented in Figure 3. The findings revealed that for both bacterial strains, no bacterial growth inhibition was promoted by E-141ii for all tested concentrations for the non-illuminated samples. Thus, E-141ii was not toxic in the dark. Additionally, the result also demonstrates that the red light only was not toxic for both bacteria because the illuminated bacterial samples containing non-E-141ii did not present a statistically significant reduction of the bacterial growth (Figure 2(C(a),D(a))). Nevertheless, E-141ii was able to photoinactivate both MSSA and MRSA strains under red light irradiation. However, these strains required different concentrations of E-141ii to inhibit bacterial growth (Figure 3). While the aPDI effect of E-141ii caused a 2.1-log unit reduction in CFU/mL at 1.0 µM and was able to inhibited to the detection limit of the method the MSSA growth at concentrations over 1.0 µM (in the 2.5 to 20 µM range), a log reduction of 1.4, 2.8, 3.4, and 3.6 was induced at 1.0, 2.5, 5.0, and 10 µM in MRSA, respectively. Nevertheless, the MRSA was totally photoinactivated at 20 µM.

López-Carballo et al. showed that E-141ii under blue light promoted a 4- and 5-log reduction in *S. aureus* and *L. monocytogenes* growth (Gram-positive strains), respectively. On the other hand, no bacterial growth reduction was observed for *E. coli* and *Salmonella* spp. [24]. Caires et al. showed that E-141ii subjected to red light efficiently inhibits the growth of *S. aureus* [5]. Nevertheless, no aPDI effect was observed on *E. coli*. However, López-Carballo demonstrated that it is possible to photoinactivate *E. coli* by using E-141ii when combining high PS concentration and white-light illumination (halogen lamp) [26]. Gram-negative bacteria are usually more resistant to aPDI than Gram-positive bacteria due to their cellular morphology, which presents an extra cell wall layer that is difficult for the PS to penetrate the cell [18]. In turn, Krüger et al. showed that *E. coli* strain NR698 (a deficient outer membrane Gram-negative strain of *E. coli*) was almost as sensitive to the aPDI effect promoted by chlorophyllin as the Gram-positive *B. subtilis* strain [30]. This result confirms that the outer membrane plays a significant role in aPDI. Josewin et al. also evaluated the application of E-141ii in association with blue illumination (LED) to inactivate *L. monocytogenes* and *Salmonella* spp. on cantaloupe rind [27]. However, they observed that the E-141ii aPDI efficacy against both bacteria was not statistically different from photoinactivation promoted by blue light alone [27]. Although either blue or red illumination could be used to excite the E-141ii molecules, it is worth stressing that we choose red light to avoid the phototoxicity of blue light [27,31]. Furthermore, red light has a better ability to penetrate biological tissues, a desirable feature for efficient photosensitization in medical applications [5].

**Figure 4:**
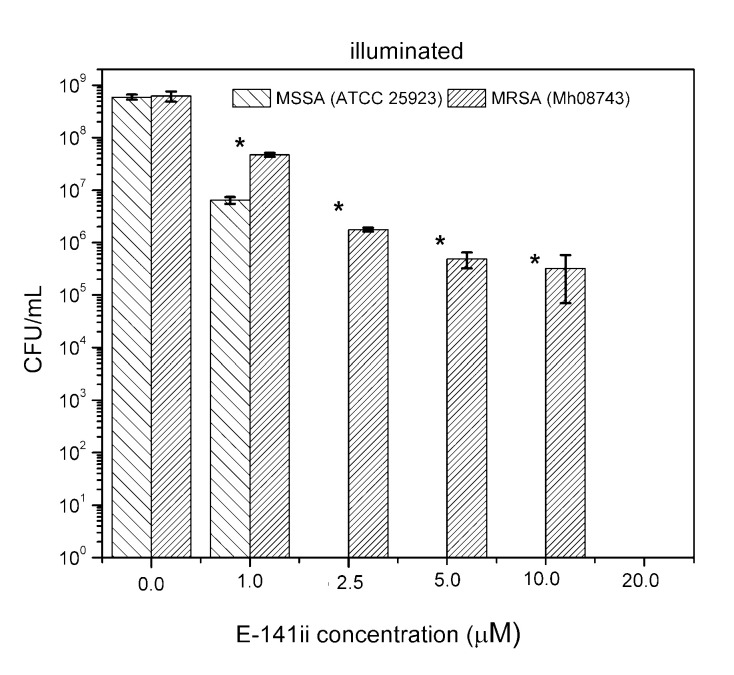
Growth response of MSSA and MRSA when subjected to different E141ii concentrations under red-light illumination at 625 nm for 1 h (30 J cm^−2^). * indicates a significant difference (paired sample *t*-test, *p* < 0.05) between the sample groups when compared at a fixed E-141ii concentration. Error bars represent the standard deviation.

Although few studies have indicated the potential use of E-141ii to promote aPDI of *S. aureus*, its capability of acting successfully as a PS against an MRSA strain is demonstrated here for the first time. Despite demanding a higher concentration to photoinactivate the MRSA, the present findings demonstrated that the E-141ii can be applied to efficiently photoinactivate both MRSA and MSSA under red-light illumination as successfully applications using E-141ii and other chlorophyll derivatives typically apply concentrations in the 1.0 to 100.0 µM range, depending on the light dose and wavelength [23,24,25,26,27,28,29,30,32,33]. We also verified that MRSA was less susceptible to aPDI than MSSA at *p* < 0.05 (paired sample *t*-test) as presented in Figure 4, requiring a higher concentration of E-141ii to inhibit bacterial growth. This observation agrees with previous findings presented by Grinholc et al. [34], which determined that MRSA strains are less responsive than MSSA to aPDI by evaluating more than 400 clinical samples of MRSA and MSSA. However, they did not find any plausible mechanism of the strain-dependent response to aPDI by trying to correlate the antibiotic susceptibility with intracellular and extracellular bacterial proteins [34]. In general, other studies have demonstrated that a multidrug sensitive strain is more susceptible to aPDI when compared with its multidrug-resistant strain [35,36]. By contrast, there is a study reporting that aPDI similarly inactivates multidrug-sensitive and multidrug-resistant strains of the same bacterial species, regardless of drug resistance [37]. Notwithstanding, the mechanisms underlying strain-dependent response to aPDI remain unclear [34,35].

It is important to highlight that E-141ii (sodium copper chlorophyllin) is more resistant and stable to light, acid, and heat when compared with other chlorophyll derivatives [20,32,38], and it is widely employed in cosmetic and medicinal products as an additive or dye, in addition to its application as food colorant [38]. However, despite its potential as a PS for photoinactivation, metal-free sodium chlorophyllin (E-140ii) is the chlorophyll derivative most applied in aPDI. The rare use of E-141ii in aPDI may be related to a premise that E-141ii could not work as a PS because of the lack of long-lived excited state due to the presence of the paramagnetic Cu^2+^ in its chemical structure [23]. Sciuti et al. reported that E-141ii does not have an electronic population in long-lived excited states, as a triplet state, and has no fluorescence, exhibiting a rapid deexcitation of the first excited singlet state (~25 ps) due to the internal conversion process [39]. Nonetheless, all these features did not preclude E-141ii acting as a PS, as demonstrated here. Even so, its aPDI mechanisms are not yet well understood.

**Figure 5:**
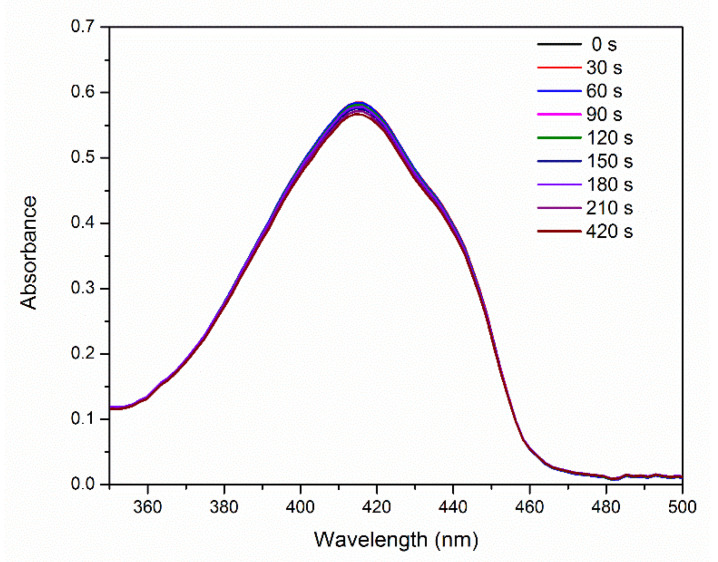
UV-vis absorption of DPBF in the presence of E-141ii as a function of the red-light illumination time.

Figure 5 shows the absorption spectra of 1,3-diphenylisobenzofuran (DPBF) in the presence of E-141ii as a function of the 625 nm light illumination time. These spectra reveal thatE-141ii did not produce ^1^O_2_ under light exposure (Type II reaction) [40] because DPBF is an optical probe that has its absorbance and fluorescence quenched when reacting with ^1^O_2_ [41,42]. This result was expected as it is well-known that E-141ii does not present an excited triplet state, a needed long-lifetime state to allow ^1^O_2_ formation by energy transfer from the excited PS (^3^PS*) to ^3^O_2_. The reliability of this protocol to assess the ^1^O_2_ production was evaluated using methylene blue (MB), a well-established PS that produces ^1^O_2_ [43]. The data demonstrate that DBPF in the presence of MB rapidly degraded under red-light illumination due to the generation of ^1^O_2_ (Figure S1 in the Supplementary Material). Therefore, our data confirm that E-141ii is not capable of inducing the aPDI via Type II reaction mechanism. This result agrees with the observed by Uchoa et al. [23], which showed by a phosphorescence detection method that E-141ii does not produce ^1^O_2_.

**Figure 6:**
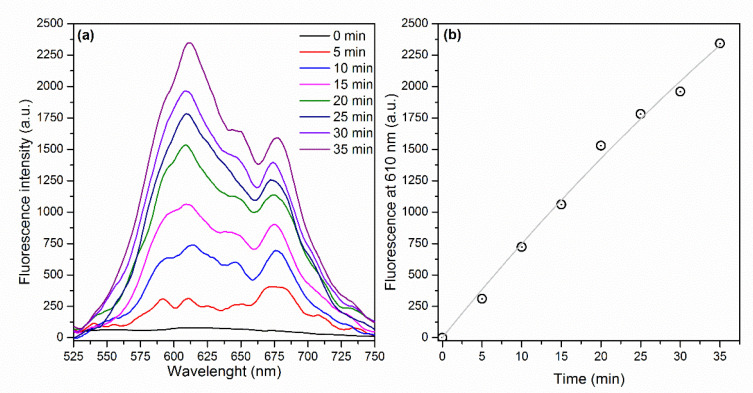
(**a**) Fluorescence spectra of DHE in the presence of E-141ii and (**b**) fluorescence intensity at 610 nm over the illumination time. The gray line represents the fitting curve obtained by using Equation (1) (R^2^ = 0.9937).

By contrast, Figure 6a displays an increase of the fluorescence signal as a function of illumination time as a consequence of the ROS generation by E-141ii (Type I reaction). Dihydroethidium (DHE) is a non-fluorescent molecule that produces a fluorescent molecule (ethidium) when interacting with different ROS species, such as superoxide (O_2_^−^), hydrogen peroxide (H_2_O_2_), peroxynitrite (ONOO^−^), and hypochlorous acid (HOCl) [44]. Figure 6b shows that a correlation coefficient of 0.9937 was obtained by fitting the fluorescence intensity at 610 nm versus illumination time by using Equation (1), with $k_{f}$ = 1.61 × 10^−4^ s^−1^ (Section S2 in the Supplementary Material). As $k_{f}$ = $k_{\mathrm{ROS}}$ [DHE] and [DHE] = 2 μM, E-141ii promoted a $k_{\mathrm{ROS}}$ of 80.5 M^−1^ s^−1^, which is in the range value usually found for the rate constant of reactions of H_2_O_2_ and O_2_^−^ with various molecules [45]. The results suggest that E-141ii induced aPDI by producing ROS for the electron transfer from its first singlet excited state to oxygen molecules (Type I mechanism).

**Figure 7:**
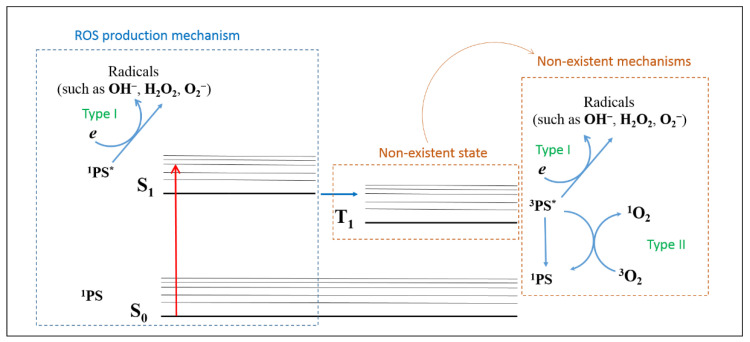
ROS production mechanism by E-141ii. 1PS, ^1^PS*, and ^3^PS* represent the PS in the ground singlet state (S_0_), first excited singlet state (S_1_), and excited triplet state (T_1_), respectively.

The present findings suggest that E-141ii can be effectively applied as a PS due to its capability of killing MRSA under red illumination by photodynamic inactivation through the Type I reaction mechanism from the first singlet excited state. As schematically shown in Figure 7, E-141ii does not present an excited triplet state (T_1_) due to the paramagnetic Cu^2+^ in its chemical structure so that the photoinactivation mechanisms Type I and Type II from the T_1_ can be ruled out. Nevertheless, the E-141ii photoinactivation mechanism can be induced by promoting the E-141ii electron from the ground singlet state (S_0_) to the first excited singlet state (S_1_) through the red-light illumination, followed by its transfer to O_2_ and production of radical species.

## 3. Materials and Methods

### 3.1. Photoinactivation Assay

The food colorant E-141ii was purchased from Sigma-Aldrich (São Paulo, Brazil). Its efficacy in aPDI was tested against an MSSA strain (ATCC 25923, Bioscan, Itu, Brazil)) and a clinically isolated MRSA strain (GenBank accession number Mh087437, HU, Campo Grande, Brazil) under red-light illumination. The strains were maintained at −70 °C in Müller-Hinton broth containing glycerol (20% *v*/*v*, QEEL, São Paulo, Brazil). The bacterial suspensions were prepared with 40 μL of the bacterial strain added to 4 mL of Müller-Hinton broth and incubated for 24 h at 37 °C. After that, E-141ii was diluted in 2 mL of a physiological saline (0.9% NaCl, Sorimax, Campo Grande, Brazil) solution containing the bacterial inoculum at 1.5 × 10^8^ CFU/mL. The E-141ii concentrations were 0.0 (negative control), 1.0, 2.5, 5.0, 10.0, and 20.0 µM. Following, the samples were shaken at 120 rpm for 30 min. After E-141ii incubation, the samples were divided into two groups: illuminated and non-illuminated. The samples of the illuminated group were placed in a 96-well plate (200 µL/well) and submitted to 625 nm light for 1 h at the dose of 30 J cm^−2^ using light-emitting diodes (LEDs, Homemade, Dourados, Brazil). Finally, for both illuminated and non-illuminated samples, a serial dilution was performed until 1:32. The total bacteria number was determined by the spread plate method using the plate count agar (PCA; Neogen Corp., Michigan, USA) medium and the colony-forming units (CFU) were counted 18 h after incubation at 37 °C. All experiments were performed in triplicate. Quantitative and statistical analyses were carried out using the Origin 8.5 software, considering the repetitions, PS concentrations, and light exposure conditions (illuminated and non-illuminated). The CFU/mL values were submitted to analysis of variance and the comparisons of the means using Student’s *t*-test with a confidence level of 95% (*p* < 0.05) for paired samples.

#### O_2_ and ROS Generation Assays

DPBF and DHE were purchased from Sigma-Aldrich (São Paulo, Brazil) and used as optical markers to investigate the photoinactivating potential of E-141ii via Type I and Type II photochemical pathways.

DPBF (2.2 mL) at 90 μM in DMSO was mixed with 0.4 mL of E-141ii at 30 μM. This solution was placed under 625 nm illumination at 3.5 mW. UV-vis absorption spectrum was recorded every 30 s in a LAMBDA 265 UV-vis spectrophotometer (Perkin Elmer, Boston, MA, USA). The ^1^O_2_ production was evaluated using the protocol adapted by Pivetta and collaborators [42] by monitoring the degradation of DPBF promoted by its interaction with ^1^O_2_. In turn, 2.0 mL of E-141ii at 30 μM in DMSO was mixed with 4 μL of DHE at 1 mM to determine the ROS production via the Type II mechanism. This solution was exposed to 625 nm illumination (12 mW), and the fluorescence spectrum was collected every 5 min using 500 nm excitation (FluoroMate FS-2, Scinco, Seoul, Korea). ROS generation was determined by following the increase of the fluorescence intensity in the 525–750 nm range. The rate constant of ROS production was estimated by kinetic analysis of the fluorescent products generated by the interaction between DHE and ROS. DHE was used at a saturating concentration (2 μM) for the fluorescence measurements, and it was assumed the formation rate of new fluorescent products *[F]* equal to the one of ROS generation by E-141ii under illumination: $DHE+ROS\;\overset{k}{\to }\;F$ [46]. Consequently, the rate of ROS production is written as $-\frac{d\left[ROS\right]}{dt}=k_{ROS}\;\left[DHE\right]\left[ROS\right]$ where $k_{\mathrm{ROS}}$ is the apparent rate constant of ROS production (i.e., the apparent rate constant for the reaction of DHE with ROS), with *[ROS]* ∝ *F*. Therefore, the ROS production equation can be rewritten as $\;-\frac{dF}{dt}=k_{f}\;F$, with $k_{f}=k_{ROS}\left[DHE\right]$, which leads to Equation (1).

(1) $$F=a\left(1-e^{-k_{f}t}\right)$$

## 4. Conclusions

The present study showed that the food colorant E-141ii can be applied to photoinactivate MRSA under red-light illumination. Nevertheless, MRSA was less susceptible than MSSA to the aPDI process, requiring a higher E-141ii concentration to inhibit bacterial growth. Additionally, the findings point out that E-141ii can be effectively applied as a PS for being able to inactivate bacteria through Type I photodynamic reaction mechanism by producing ROS due to the electron transfer from the first singlet excited state of E-141ii to oxygen molecules.

## Supplementary Materials

The following are available online. Figure S1: (**a**) UV-vis absorption spectra and (**b**) absorbance at 415 nm of DPBF in the presence of methylene blue as a function of the red-light illumination time.
