# Supplementary material for: Photodynamic Inactivation of Methicillin-Resistant Staphylococcus aureus by a Natural Food Colorant (E-141ii)
Source: Molecules. 2020 Sep 29;25(19):4464. doi: 10.3390/molecules25194464 (PMC7582792; doi:10.3390/molecules25194464)
Supplement: Supplementary file 1 [file molecules-25-04464-s001.pdf]

molecules

Supplementary material

# Photodynamic Inactivation of Methicillin-Resistant *Staphylococcus aureus* by a Natural Food Colorant (E-141ii)

**Cynthia S.A. Caires [1,2], Cicera M. Silva [3], Alessandra R. Lima [3], Lurian M. Alves [3], Thalita H.N. Lima [3], Ana C.S. Rodrigues [1], Marilene R. Chang [1], Samuel L. Oliveira [3], Corinne Whitby [2], Valter A. Nascimento [1,*] and Anderson R.L. Caires [2,3,*]**

[1] Graduate Program in Health and Development in the Midwest Region, Faculty of Medicine, Federal University of Mato Grosso do Sul, Campo Grande 79070-900, Brazil; cynthiasuzyelen@yahoo.com.br (C.S.A.C.); anaclaurod@yahoo.com.br (A.C.S.R.); marirchang@yahoo.com.br (M.R.C.)
[2] School of Life Sciences, University of Essex, Colchester CO4 3SQ, UK; cwhitby@essex.ac.uk
[3] Optics and Photonics Group, Institute of Physics, Federal University of Mato Grosso do Sul, Campo Grande 79070-900, Brazil; ciceraquimica@gmail.com (C.M.S. ); ramos.alessandra.09@gmail.com (A.R.L.); lurianalves@gmail.com (L.M.A.); thalita.lima212@gmail.com (T.H.N.L.); samuel.oliveira@ufms.br (S.L.O.)
* Correspondence: valter.aragao@ufms.br (V.A.N.); anderson.caires@ufms.br (A.R.L.C.)

Academic Editor: M. Amparo F. Faustino
Received: 27 July 2020; Accepted: 28 August 2020; Published: date

## S1. $^1O_2$ generation by Methylene blue

2.2 mL of DPBF at 90 µM in DMSO was mixed with 0.4 mL of methylene blue at 30 µM. This solution was placed under 625-nm illumination at 3.5 mW. UV-Vis absorption spectrum was collected every 30 s in a LAMBDA 265 UV/VIS spectrophotometer (Perkin Elmer). The $^1O_2$ generation was evaluated by monitoring the DPBF degradation caused by its interaction with $^1O_2$ [1].

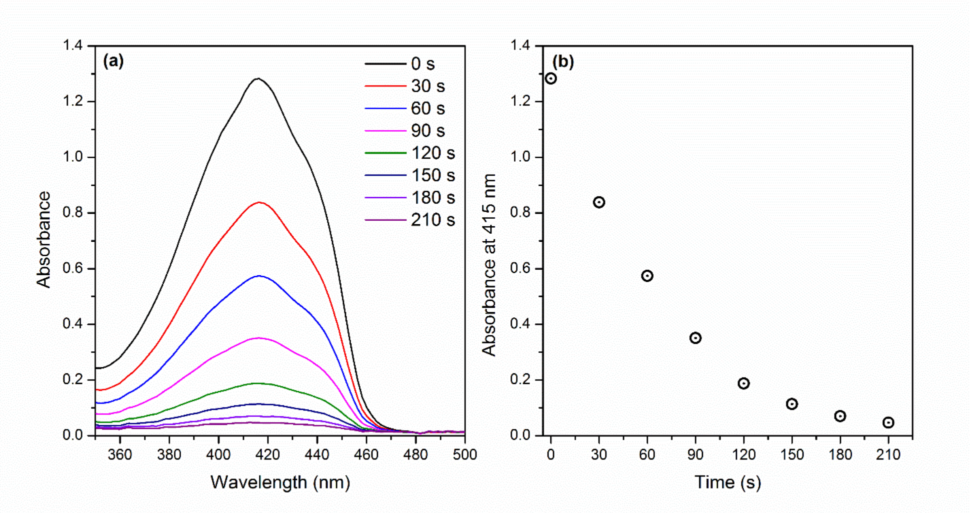


**Figure S1.** (a) UV-Vis absorption spectra and (b) absorbance at 415 nm of DPBF in the presence of methylene blue as a function of the red-light illumination time.

## S2. ROS production kinetic analysis

The ROS production rate constant was determined by analyzing the kinetic of the fluorescent products generated by the interaction between ROS and DHE. A saturating concentration of DHE was used during the measurements, and it was assumed that the formation rate of new fluorescent products [F] was equal to the one for ROS produced by E-141ii under red light illumination, as follows [2]:

$$\boldsymbol{DHE} + \boldsymbol{ROS} \overset{k}{\rightarrow} \boldsymbol{F} \quad \text{Eq. (S1)}$$

Consequently, the rate of ROS generation can be written as:

$$-\frac{d[\boldsymbol{ROS}]}{dt} = \boldsymbol{k_{ROS}}\,[\boldsymbol{DHE}][\boldsymbol{ROS}] \quad \text{Eq. (S2)}$$

where $\boldsymbol{k_{ROS}}$ is the rate constant of ROS production, with $[\boldsymbol{ROS}] \propto \boldsymbol{F}$. Eq. S2 can be expressed as:

$$-\frac{dF}{dt} = \boldsymbol{k_f}\,\boldsymbol{F} \quad \text{Eq. (S3)}$$

where $\boldsymbol{k_f} = \boldsymbol{k_{ROS}}[\boldsymbol{DHE}]$. From Eq. S3, the following equation can be obtained.

$$\boldsymbol{F} = \boldsymbol{a}(\boldsymbol{1} - \boldsymbol{e}^{-\boldsymbol{k_f t}}) \quad \text{Eq. (S4)}$$

Where *F* is the fluorescence intensity originated from the reaction of DHE with the ROS produced under red light illumination, and *t* is the illumination time, and $\boldsymbol{k_f}$ and $\boldsymbol{a}$ are constants that can be determined by fitting the experimental data presented in Figure S3 by using Eq. S4.

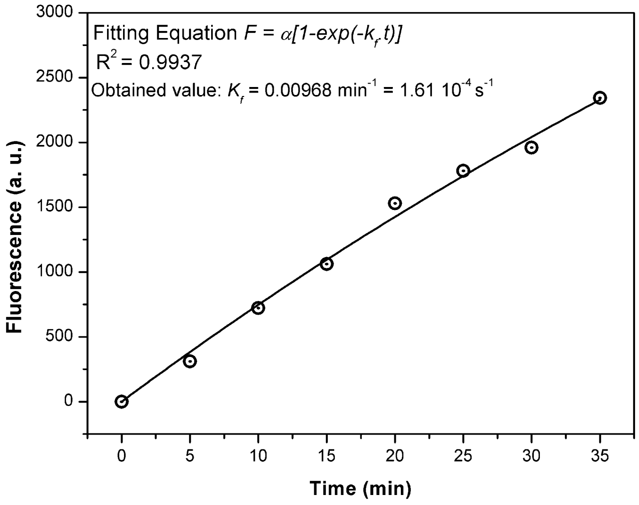


Figure S2. Fluorescence intensity at 610 nm as a function of the illumination time. The gray line represents the fitting curve obtained by using Eq. S4.

Finally, the value of $k_{ROS}$ as 80.5 $M^{-1}$ $s^{-1}$ was determined by knowing that $[DHE]$ = 2 μM and $k_f = 1.61\ 10^{-4}\ s^{-1}$.


1. Pivetta, R.C.; Auras, B.L.; Souza, B. de; Neves, A.; Nunes, F.S.; Cocca, L.H.Z.; Boni, L. De; Iglesias, B.A. Synthesis, photophysical properties and spectroelectrochemical characterization of 10-(4-methyl-bipyridyl)-5,15-(pentafluorophenyl)corrole. *J. Photochem. Photobiol. A Chem.* **2017**, *332*, 306–315, doi:10.1016/j.jphotochem.2016.09.008.

2. Zhao, H.; Kalivendi, S.; Zhang, H.; Joseph, J.; Nithipatikom, K.; Vásquez-Vivar, J.; Kalyanaraman, B. Superoxide reacts with hydroethidine but forms a fluorescent product that is distinctly different from ethidium: potential implications in intracellular fluorescence detection of superoxide. *Free Radic. Biol. Med.* **2003**, *34*, 1359–1368, doi:10.1016/S0891-5849(03)00142-4.
